# Long-term survival with anlotinib as a front-line treatment in an elderly NSCLC patient: A case report

**DOI:** 10.3389/fonc.2023.1043244

**Published:** 2023-04-06

**Authors:** Jingyi Wang, Xiaoqing Li, Juan Zhou, Dan Qiu, Mengyao Zhang, Lan Sun, Shengwen Calvin Li

**Affiliations:** ^1^ Department of Oncology, Bishan Hospital of Chongqing Medical University, Chongqing, China; ^2^ Neuro-Oncology and Stem Cell Research Laboratory, Center for Neuroscience Research, CHOC Children’s Research Institute, Children’s Hospital of Orange County (CHOC), Orange, CA, United States; ^3^ Department of Neurology, University of California-Irvine School of Medicine, Orange, CA, United States

**Keywords:** non-small cell lung cancer, anti-angiogenesis therapy, front-line treatment, elderly patients, case report, Anlotinib hydrochloride, tegafur-uracil, chemotherapy

## Abstract

**Background:**

Half of the population of non-small cell lung cancer (NSCLC) patients are older than 70 years and have limited therapeutic options due to poor tolerance and being excluded in most clinical trials. Anlotinib hydrochloride, a novel oral multi-target tyrosine kinase inhibitor, has been approved for the standard third-line treatment for NSCLC in China. Herein we report an elderly NSCLC patient without any driver gene mutations who was undergoing anlotinib as a front-line treatment and who achieved long-term survival.

**Case summary:**

The 77-year-old male patient was admitted to the hospital for chest tightness after engaging in physical activity for a week. The patient has been diagnosed with stage IIIB driver gene-negative squamous cell lung carcinoma. After that, he was treated with anlotinib for 2 years and 10 months from the first diagnosis until the last disease progression. Briefly, anlotinib combined with platinum-based chemotherapy was performed as the first-line therapy over six cycles. After 6 more cycles of anlotinib monotherapy maintenance, disease progression occurred. Then, anlotinib combined with tegafur was administered as a salvage treatment, and the disease was controlled again. After 29 cycles of anlotinib combined with tegafur regimens, the disease progressed finally. The patient achieved a total of 34 months of progression-free survival after anlotinib was used as the front-line treatment. He is still alive with a good performance status now (performance status score: 1).

**Conclusion:**

This patient achieved long-term survival using anlotinib as a front-line regimen combined with chemotherapy.

## Introduction

Lung cancer is one of the most common malignant tumors and the leading cause of global cancer mortality (1). Angiogenesis is one of the characteristics of malignant tumors, which can provide nutrition for the growth of tumor cells and secrete growth factors to promote cancer cell proliferation, thus playing an essential role in tumor growth, invasion, and metastasis (2, 3). A growing number of studies have shown that advanced non-small cell lung cancer (NSCLC) patients can benefit from anti-angiogenesis therapy with higher anti-cancer activity and fewer adverse effects than traditional chemoradiotherapy (4, 5). Anlotinib is a novel oral multitarget tyrosine kinase inhibitor which strongly inhibits vascular endothelial growth factor (VEGF), platelet-derived growth factor receptor (PDGF), fibroblast growth factor receptor (FGFR), and stem cell factor receptor (c-Kit), resulting in the inhibition of the growth of tumor blood vessels (6). On May 9, 2018, anlotinib was approved by China Food and Drug Administration as a standard third-line treatment regimen for advanced NSCLC based on the results of the ALTER-0303 study (7). However, the evidence for anlotinib as a front-line treatment for NSCLC is limited. Herein we report an elderly NSCLC patient without any driver gene mutations undergoing anlotinib as a front-line treatment.

## Background

### Chief complaints

A 77–year-old male patient developed chest tightness after an activity and precardiac pain in June 2018. He had an occasional cough with sputum.

### History of present illness

The patient had chest tightness after an activity, precardiac pain, and occasional cough with sputum.

### History of past illness

The patient had a history of chronic obstructive pulmonary disease (COPD) and diabetes mellitus type 2 (T2DM), with a smoking history of 55 years averaging 20 cigarettes per day. He had quit smoking for more than 2 years.

### Personal and family history

The patient had a cancer-relative family susceptibility. His one brother and two sisters suffered from lung cancer, and another brother had liver cancer.

### Physical examination

The results of the physical examination showed that the patient’s body temperature was 36.3°C, the pulse rate was 70 beats per minute (bpm), the blood pressure was 128/76 mmHg, respiratory rate was 19 breaths/min, and the performance status score was 1.

### Laboratory examinations

His blood count showed a WBC level of 5.75 × 10^9^/L, Hb level of 116 g/L, and platelet count of 201 × 10^9^/L. The serum level of carcinoembryonic antigen was 6.6 ng/ml.

### Pathology and genetic testing

**Figure 1:**
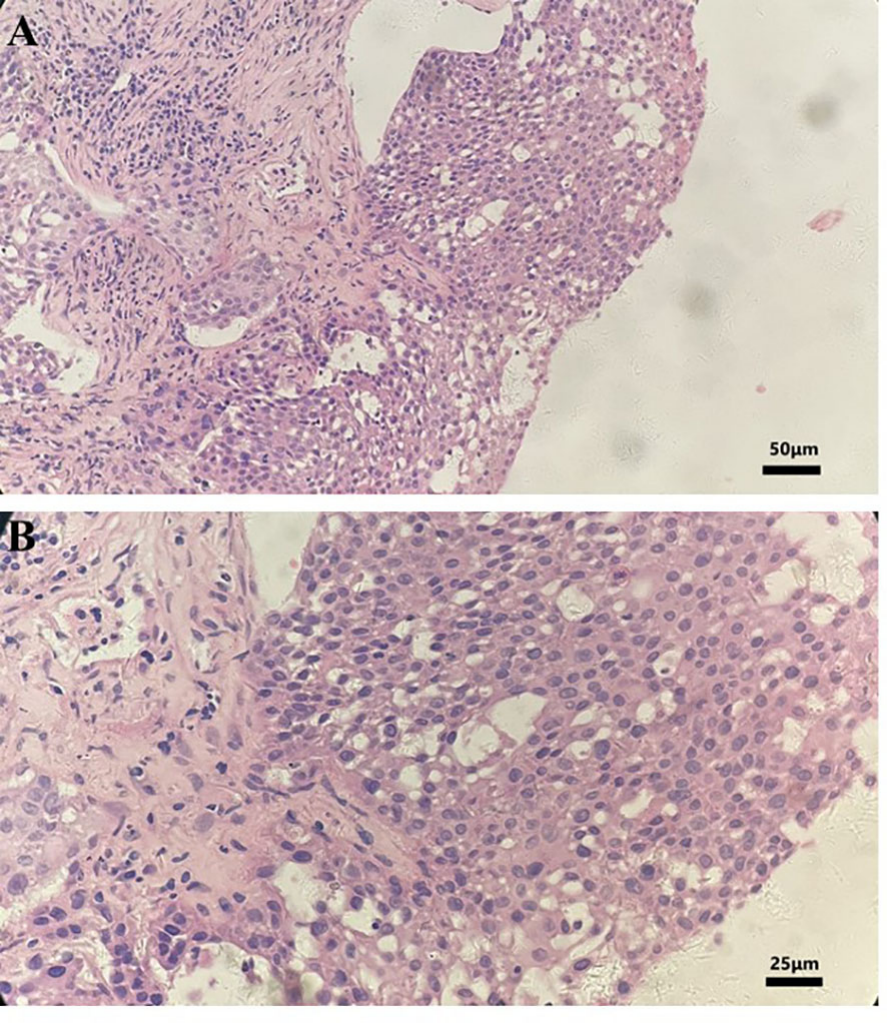
Hematoxylin and eosin staining photomicrographs of a right lung tumor tissue biopsy. **(A)** Magnification, ×200. **(B)** Magnification, ×400.

The histopathological analysis of the tissue biopsy samples collected from the right lung revealed poorly differentiated squamous cell carcinoma (**Figure 1**). The immunohistochemistry result showed the following details: CK5/6 (+), P63 (+), P40 (+), NapsinA (-), TTF1 (-), CK7(-), and CK14(+). A molecular analysis did not find any driver gene mutations of EGFR, ALK, and ROS1. Tissue samples were detected *via* next-generation sequencing with a panel consisting of 211 genes, which revealed TP53 (mutant abundance: 14.37%) and tumor mutation burden (TMB) of 21.15 Muts/Mb. The somatic mutations are shown in **Table 1**.

**Table 1:** List of somatic mutations.

Gene	Exon	Nucleotide variation	Amino acid change	Mutation abundance
PIK3CA	exon10	c.1624G>A	p.E542K	1.14%
TP53	exon6	c.637C>T	p.R213*	14.37%
FANCM	exon20	c.5237C>G	p.S1746*	1.18%
PTEN	exon6	c.631T>C	p.C211R	9.78%
PALB2	exon4	c.1366G>C	p.E456Q	8.04%
ATR	exon3	c.157G>C	p.V53L	4.35%
GATA3	exon3	c.388C>T	p.L130F	23.42%
HNF1A	exon2	c.331G>A	p.D111N	12.28%
JAK2	exon22	c.2951T>G	p.V984G	11.49%
NTRK3	exon14	c.1540C>A	p.P514T	10.60%
MAP3K1	exon1	c.212A>T	p.D71V	1.50%

The asterisk symbol (*) indicates the ending amino acid position during translation upon a stop codon (or termination codon) that is a codon for transcription termination signal. The single letter is for the abbrreviation of amino acids and the number is for the position of an amino acid) in the "amino acid change" column.

### Imaging examinations

**Figure 2:**
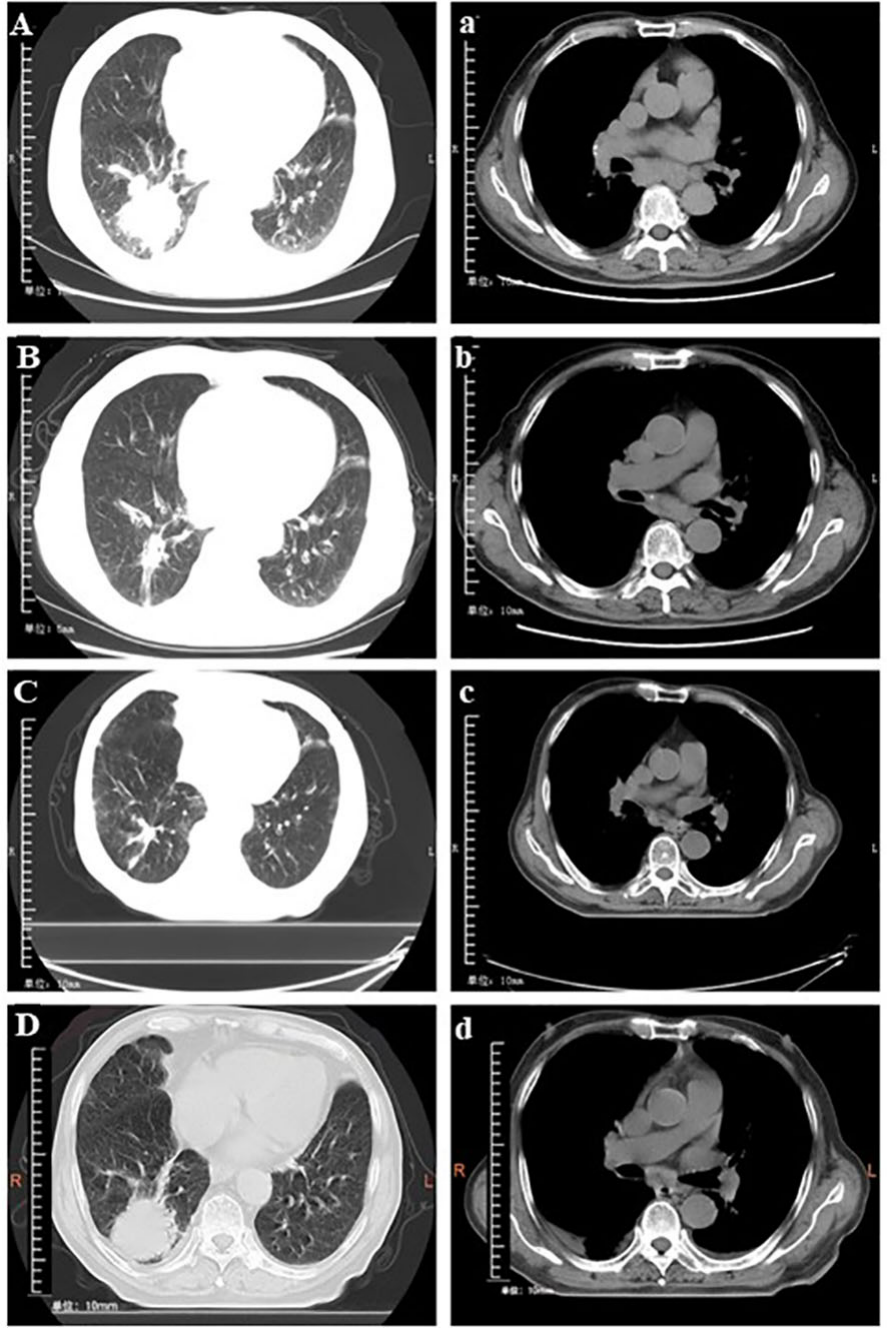
Conversion of chest computed tomography at different times. (**A** and **a**) Baseline findings on chest computed tomography (tumor size: 52 × 55 mm). (**B** and **b**) The mass shrank significantly (tumor size: 31 × 18 mm) after treatment with four cycles of anlotinib plus cisplatin. (**C** and **c**) Stable disease (tumor size: 25 × 21 mm) following 12 cycles of anlotinib + tegafur. (**D** and **d**) Progressive disease (tumor size: 41 × 34 mm) after 29 cycles of anlotinib + tegafur.

The chest scan first computed tomography (CT) result showed a mass which was 52 mm × 50 mm in size (**Figure 2A**). No metastasis was observed on enhanced CT of the skull and the abdomen. The lymph node ultrasound showed no metastasis. As per the solid tumor version 1.1 (RECIST 1.1) response evaluation criteria, the maximum lesion reduction reached 40.4% after four cycles of anlotinib with platinum-based chemotherapy (**Figure 2B**). After six courses of anlotinib monotherapy maintenance, the MRI results showed bilateral clavicular lymph node metastasis, and the patient’s PFS_1_ was 10 months. Then, after 12 cycles of anlotinib in combination with tegafur, the lesion reduction reached 13.8% compared with the previous best curative effect (**Figure 2C**). The disease remained stable after this, and there were no significantly enlarged lymph nodes on bilateral neck ultrasound. On May 24, 2021, the lesion enlarged by 41.4%, and the efficacy was evaluated as disease progression (**Figure 2D**), so the patient’s PFS_2_ was 24 months.

### Final diagnosis

Finally, the patient was diagnosed with stage IIIB poorly differentiated driver gene-negative squamous cell lung carcinoma (cT3N2M0 in accordance with version 8 of TNM staging).

### Treatment

**Figure 3:**
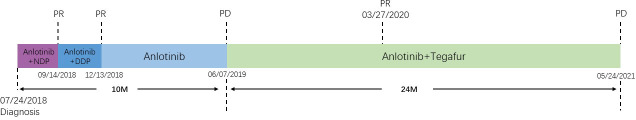
Timeline of events from diagnosis to progressive disease and summary of the administered treatments. Treatment work flow from [(anlotinib + NDP) à (anlotinib + DDP)] (6 cycles) to(anlotinib) (6 cycles) and to (anlotinib + tegafur) (29 cycles). After undergoing this treatment strategy, the patient has achieved a PFS of 34 months and an OS of 53 months. These results surpass those seen in the majority of real-world studies and clinical trials.

Since July 24, 2018, the patient was treated with anlotinib (12 mg, d1–d14, q3w) and nedaplatin (85 mg/m^2^, q3w) for the first-line therapy. After six courses of anlotinib combined with platinum-based chemotherapy, we used anlotinib monotherapy for maintenance treatment (12 mg, d1–d14, q3w), but the patient reduced the drug dose on his own during the last two courses of maintenance therapy. The disease progressed after six cycles of maintenance treatment. Afterwards, the regimen was switched to anlotinib (12 mg, d1–d14, q3w) combined with tegafur (120 mg d1–d14, q3w) (**Figure 3**).

### Outcome and follow-up

As of May 24, 2021, the patient received 2 years and 10 months of anlotinib treatment, with a progression-free survival of 34 months. The degree of adverse event was assessed according to Common Terminology Criteria Adverse Events Version 5.0. During the period of the treatment of anlotinib combined with nedaplatin, the patient experienced thrombocytopenia in the second cycle (grade 4), which was controlled with thrombopoietin therapy. Fatigue (grade 2) developed during the 11th and 12^th^ cycles, which gradually improved during the interval between cycles. After the disease progressed, we proposed to adjust the treatment option several times, but the patient and his family refused. The last follow-up was on December 12, 2022, and the CT result showed that the lesion size was 78 mm × 59 mm, which increased from the original diagnosis of 52 mm × 50 mm in size (**Figure 2A**).

## Discussion

In this case, the patient was diagnosed in July 2018 (cT3N2M0), and he could first be considered for surgery. However, his family adamantly refused due to his advanced age and underlying diseases. We also considered immunotherapy in combination with platinum-based chemotherapy, but PD-1 inhibitors were expensive and not financially affordable the patient and his family. Shortly before his diagnosis, anlotinib was approved by China Food and Drug Administration as a third-line treatment for advanced squamous lung cancer (the peripheral type only) based on the results of two clinical trials of anlotinib as a third-line or further treatment for NSCLC. One clinical study (ALTER0302) showed that the median progression-free survival (PFS) of the anlotinib group was significantly better than that of placebo (4.8 vs. 1.2 months) (8). The other clinical trial (ALTER0303) showed that anlotinib has extended median OS and PFS significantly, that is, 9.6 months vs. 6.3 months and 5.4 months vs. 1.4 months, respectively, compared with the placebo. Under the third-line or beyond treatment setting, the anlotinib combination therapy showed manageable toxicities and encouraging efficacy, indicating a good application prospect (9), which is one of the reasons why we administrated anlotinib as a front-line treatment. The other reasons are as follows: first, according to the National Comprehensive Cancer Network guidelines (2018 edition), radical concurrent chemo-radiotherapy is preferred for inoperable stage IIIB NSCLC patients. However, clinical studies have shown that concurrent radiotherapy is poorly tolerated in elderly patients (10), with a high possibility of discontinuing treatment. Sequential chemoradiotherapy may be considered for patients who cannot tolerate concurrent chemoradiotherapy. However, this patient had multiple underlying diseases such as COPD and T2DM; thus, the risk of uncontrollable side effects was high. Moreover, the target volume was too large to determine an appropriate radiotherapy plan ensuring antitumor efficacy and safety. Third, the toxicity of anti-angiogenesis drugs, including anlotinib, is much lower than chemotherapy, and adverse effects are controllable (9, 11). Anlotinib is reported to have the advantage of low toxicity (12). The most frequent toxicities include hypertension, hand–foot syndrome, fatigue, *etc.* Fourth, anti-angiogenesis treatment required attention to the side effect of hemoptysis. Fortunately, this patient had no symptoms of active hemorrhage, and the lesion was peripheral, so the risk of hemorrhage was evaluated as low. Fifth, the patient and his family refused to undergo radiation therapy, so we applied to the Ethics Committee for the inclusion of anlotinib as first-line treatment. The patient and his family eventually chose anlotinib as the first-line treatment and signed the informed patient’s consent as approved. He achieved a total progression-free survival of 34 months using anlotinib as the front-line regimen. From the time of the patient’s diagnosis to the time of the last follow-up (46 months), the maximum diameter of his tumor lesion increased by 12 mm. No uncontrollable toxic side effects were observed in this patient during the drug administration. We analyzed this patient’s long-term survival for several reasons. Firstly, this patient did not develop distant metastases, and the clinical studies associated with anlotinib have validated distant metastases as an important prognosis (13). Secondly, it has been shown that inhibition of autophagy improves the efficacy of anlotinib (14), and age is one of the important reasons for the effect of autophagy (15). Given the advanced age of this patient, we consider that he may have a sustainable survival benefit due to his autophagy inhibition status. Thirdly, this patient has hypofractionated squamous carcinoma. Although hypofractionated tumor cells are conventionally more malignant, there is a complex relationship between cancer and inflammation. Hypofractionated cell proliferation may inhibit the growth of tumor cells through specialized pro-resolving mediators (16), which may also be a factor in this patient’s long survival. Fourthly, the patient in this case had TP53 mutations (mutant abundance: 14.37%) with TMB: 21.15 Muts/Mb. TP53 mutations have been identified to be involved in the process of neovascularization associated with increased VEGF expression, which is one of the important targets of anlotinib. Fu et al. found that TP53 mutations are significantly associated with a favorable prognosis in patients with advanced solid malignancies (17). It has also been shown that advanced NSCLC patients with high TMB mutations (>10 Muts/Mb) can benefit from anti-angiogenesis therapy (18). Unfortunately, we could not ascertain PD-L1 expression because of the insufficient volume of tissue obtained in the first biopsy and the patient’s unwillingness to undergo a repeat biopsy.

This patient had disease progression after six cycles of anlotinib monotherapy maintenance. We switched to anlotinib combined with tegafur because oral tegafur treatment was more convenient and economical. The EAST study demonstrated that tegafur is equally as efficacious as docetaxel (19). In addition, anti-angiogenesis therapy can improve the local hypoxic condition of the tumor microenvironment, which is more conducive to the entry of chemotherapeutic drugs into the tumor tissue. There is a growing number of studies on anlotinib as a second-line treatment from 2018 onwards. One study showed that anlotinib combined with chemotherapy might be an effective and well-tolerated treatment for advanced NSCLC in patients who fail in first- or second-line therapy (20). Anlotinib plus camrelizumab had shown promising efficacy and manageable toxicity as a second-line or later-line therapy for NSCLC, especially in the 12 mg cohorts (21). However, another study showed that anlotinib was less effective than platinum-pemetrexed chemotherapy in T790M-negative lung adenocarcinoma, implicating that it may be more suitable for squamous cell lung carcinoma (22). Hence, anlotinib has a synergistic antitumor effect and good safety for NSCLC and may be promising for front-line treatment for NSCLC (20). There have also been more studies of anlotinib as a first-line treatment in the last 2 years, and most of these studies have adopted the combination therapy model. The latest clinical analysis of first-line therapy in elderly patients with advanced lung adenocarcinoma without driver gene mutations showed similar median PFS (3.0 m vs. 2.8 m) and OS (7.0 m *vs*. 7.0 m) in the anlotinib group and the chemotherapy group (P > 0.05), and there was no significant difference in ORR (17.5 vs. 15%) or DCR (67.5 vs. 65.5%) between both treatment groups (23). The other studies have shown that, for driver gene-negative NSCLC patients, the median PFS in the anlotinib combined with chemotherapy group was 1.54 months longer than that in the chemotherapy group (9.38 months vs. 7.84 months, *P* < 0.05), and the median OS in the anlotinib combined with chemotherapy group was longer as well (11.52 months vs. 10.46 months), but the difference was not statistically significant (*P* > 0.05) (24). A study on inoperable NSCLC patients indicated that the median PFS of sequential chemoradiotherapy patients was 10.8 months, but grade 3 acute esophagitis occurred in four of 78 patients (5%) (25). As shown in **Table 2** (18, 23, 26), anlotinib is promising for the first-line treatment for NSCLC with its median PFS range of 3.0 to 15.0 months.

**Table 2:** Some clinical trials of anlotinib in non-small cell lung cancer (NSCLC) for the first line.

Line(s) of treatment	Study population	Study population	Number of patients	Median progression-free survival (months)	ORR (%)	DCR (%)	Reference
First line	Patients with EGFR wild-type stage IIIB–IV NSCLC aged more than 70 years	Anlotinib (12 mg, d1–d14/q3w)	40	3.00	17.50	67.50	(22)
First line	Patients with treatment-naïve and EGFR wild-type stage IIIB–IV NSCLC aged 18 to 75 yeas	Anlotinib (12 mg, d1–d14/q3w) + sintilimab (200 mg, d1/q3w)	22	15.00	72.70	100.00	(18)
First line	Patients with EGFR-mutated locally advanced and/or metastatic stage IIIB-IV NSCLC aged 18 to 75 years	Anlotinib (12 mg, d1–d14/q3w) + icotinib (125 mg, tid)	35	6.01	59.00	88.00	(25)

Recently, more studies focused on the first-line usage for NSCLC but less for elderly patients. To the best of our knowledge, the NSCLC patients in most clinical trials were not allowed to be older than 75 years—for example, the median age of patients with lung cancer who were treated in pivotal trials involving immunotherapy ranged from 61 and 65 years, which are lower than the median age at diagnosis. Thus, such poor representation of older patients in clinical trials makes truly evidence-based decisions on the best regimen for geriatric patients difficult. This patient was 77 years old at diagnosis and achieved a total PFS of 34 months and OS of 53 months after anlotinib treatment, which exceeded most of the results of real-world studies and clinical trials. This case report suggests that combining anlotinib with chemotherapy shows promise as a front-line treatment for elderly patients with advanced NSCLC. Further prospective studies are necessary to validate these findings.

## Conclusion

As a front-line treatment, anlotinib significantly prolonged this elderly NSCLC patient’s survival time and improved his quality of life.

## Data availability statement

The original contributions presented in the study are included in the article/supplementary material. Further inquiries can be directed to the corresponding author.

## Ethics statement

The studies involving human participants were reviewed and approved by The ethics committee of The People’s Hospital of Bishan District Chongqing. The patients/participants provided their written informed consent to participate in this study. Written informed consent was obtained from the individual(s) for the publication of any potentially identifiable images or data included in this article.

## Author contributions

JW and JZ contributed significantly to analysis and manuscript preparation. JW performed the data analyses and wrote the manuscript. XL and DQ helped perform the analysis with constructive discussions. MZ assisted in joint evaluation of patients and figure design. LS contributed to the conception of the study. SCL revised the article and made important suggestions. All authors contributed to the article and approved the submitted version.
